# *Salmonella* Immunotherapy Improves the Outcome of CHOP Chemotherapy in Non-Hodgkin Lymphoma-Bearing Mice

**DOI:** 10.3389/fimmu.2018.00007

**Published:** 2018-01-23

**Authors:** Thais Bascuas, María Moreno, Sofía Grille, José A. Chabalgoity

**Affiliations:** ^1^Laboratory for Vaccine Research, Departamento de Desarrollo Biotecnológico, Instituto de Higiene, Facultad de Medicina, Universidad de la República, Montevideo, Uruguay; ^2^Cátedra de Hematología, Hospital de Clínicas, Facultad de Medicina, Universidad de la República, Montevideo, Uruguay; ^3^Departamento Básico de Medicina, Hospital de Clínicas, Facultad de Medicina, Universidad de la República, Montevideo, Uruguay

**Keywords:** non-Hodgkin lymphoma, chemotherapy, CHOP, *Salmonella*, Immunotherapy

## Abstract

We have previously shown that *Salmonella* immunotherapy is effective to treat B-cell non-Hodgkin lymphoma (B-NHL) in mice. However, this model involves animals with high tumor burden, whereas in the clinics B-NHL patients are usually treated with chemotherapy (CHOP: cyclophosphamide, doxorubicin, vincristine, and prednisone) as first-line therapy prior to immunotherapy. Recently, we have described a NHL-B preclinical model using CHOP chemotherapy to achieve MRD in immunocompetent animals that closely resemble patients’ conditions. In this work, we assessed the efficacy of *Salmonella* immunotherapy in B-NHL-bearing mice undergoing chemotherapy. *Salmonella* administration significantly delayed tumor growth and prolonged survival of chemotherapy-treated NHL-bearing animals. Mice receiving the CHOP–*Salmonella* combined therapy showed increased numbers of tumor-infiltrating leukocytes and a different profile of cytokines and chemokines expressed in the tumor microenvironment. Further, *Salmonella* immunotherapy in CHOP-treated animals also enhanced NK cells cytotoxic activity as well as induced systemic lymphoma-specific humoral and cellular responses. Chemotherapy treatment profoundly impacted on the general health status of recipient animals, but those receiving *Salmonella* showed significantly better overall body condition. Altogether, the results clearly demonstrated that *Salmonella* immunotherapy could be safely used in individuals under CHOP treatment, resulting in a better prognosis. These results give strong support to consider *Salmonella* as a neoadjuvant therapy in a clinical setting.

## Introduction

Non-Hodgkin lymphomas (NHL) are the most frequent hemato-oncological malignancies. Current treatment for patients is a combination of chemotherapy, radiotherapy, and monoclonal antibodies, such as Rituximab (1). All combined these therapies result in high rates of complete remission; however, a substantial proportion of patients relapse with chemoresistant disease. Thus, new therapeutic options are highly necessary to improve the clinical outcome in these patients (2–4).

The use of bacteria for the development of immunotherapies for cancer has great potential (5–7). Particularly, *Salmonella* is a facultative anaerobe bacteria that can replicate and accumulate in the tumor microenvironment, which offers the potential to amplify the therapeutic effect at the tumor site, avoiding toxicity in surrounding tissues (8–10). High amount of bacteria are found in necrotic and ischemic regions, which represents an advantage for targeted immunotherapy as these areas are more resistant to radiation and chemotherapy (6, 10–12). *Salmonella* efficiency can be seen as the result of a dual effect, a direct tumoricidal activity (13, 14) and the result of a strong pro-inflammatory response elicited by the bacteria at the tumor site. *Salmonella* induces TNF-α, IFN-γ, and IL-12 production, which results in recruitment and activation of intratumoral DCs for efficient antigen presentation (15, 16). In addition, neutrophils infiltration and tumor-specific T-cell response are induced, whereas immunosuppressive cells including MDSCs and regulatory T cells (Tregs) are reduced (14, 17). Particularly, *Salmonella enterica* serovar Typhimurium (*S*. Typhimurium) has shown to be highly effective as an agent in many types of cancers in both preclinical and clinical models, with a demonstrated safety profile (18).

We developed LVR01, an attenuated strain of *S*. Typhimurium obtained by introducing a deletion into the *aroC* gene of parental *S*. Typhimurium isolate P228067 (19) and tested it as vector for heterologous antigens in different animal species and by different routes of administration. We demonstrated that LVR01 is safe and elicits strong immune responses against its own antigens as well as against the carried heterologous antigens (11, 20–23), being patented as an effective vaccine vector for deer against prion disease (U.S. Patent No. 8685718). LVR01 demonstrated also great potential as immunotherapy in the A20 mouse models of B-NHL (24, 25) as well as in 4T1 breast cancer (26) and B16 melanoma (our own unpublished results).

As most new therapies for cancer, LVR01 preclinical studies were mainly conducted as monotherapy in animals with high tumor burden. However, translation into the clinics implies evaluation in combination with standard chemotherapy (27–31). Therefore, we found it relevant to develop preclinical studies resembling the clinical trial scenario. We have recently described the feasibility of applying CHOP chemotherapy used in clinics to A20-bearing mice and demonstrated that, albeit its cytotoxic effect, CHOP chemotherapy promoted a pro-inflammatory tumor microenvironment and an immune response that resulted in an extended progression-free survival (PFS) (32). Here, we explore the feasibility and efficacy of *Salmonella* LVR01 in combination with CHOP chemotherapy to treat NHL-bearing mice.

## Materials and Methods

### Animals and Tumor Cell Line

Animal experimentation protocols were approved by the University’s Ethical Committee for Animal Experimentation, Uruguay. We use female BALB/c mice, 8–10 weeks old, which were housed on 12:12 h light/dark cycles and given food and water *ad libitum*.

The A20 cell line (American Type Culture Collection, Manassas, VA, USA) was routinely grown and maintained as previously described (33). A20 cell lysate was made from 1 × 10^7^ cells/ml by sonication, 5 pulses of 15 min in a sonicator Omni Sonic Ruptor 4000 (Omni International, Kennesaw, GA, USA). The suspension was centrifuged at maximum speed and the supernatant was kept. Protein concentration was estimated by Bradford Reagent (Sigma-Aldrich) at 2.55 mg/ml. This lysate was used for *in vitro* stimulation of splenocytes and to sensitize ELISA plates.

The B16F1 melanoma cell line was cultured in DMEM (Capricorn, Ebsdorfergrund, Germany) supplemented with 10% FBS (PAN-Biotech, Aidenbach, Germany) at 37°C in 5% CO_2_ atmosphere. This line was used to prepare a cell lysate, following the same procedure that for A20 described above.

For NK cell-mediated cytotoxicity, YAC-1 cell line (ATCC) was used. This line derives from lymphoma cells transformed with Moloney murine leukemia virus, and it is sensible to NK-mediated cytotoxicity. Cells were grown in RPMI-1640 (Sigma-Aldrich, St. Louis, MO, USA) supplemented with 10% FBS, at 37°C 5% CO_2_.

### Tumor Cells Transplantation

A20 cell line was grown in culture and harvested in log phase. Then cells were washed and resuspended to a final concentration of 5 × 10^6^ cells/ml in PBS. Mice (*n* = 10) were injected subcutaneously (s.c.) into the right flank with 1 × 10^6^ cells in 0.2 ml of PBS as we described previously (33). Tumors were measured every other day with a microcaliper and tumor volumes were calculated as length × width × depth × π/6 (11). Euthanasia was carried out by cervical dislocation when tumors reached 4,000 mm^3^ or earlier if animals showed signs of distress.

### Chemotherapy Treatment

Cyclophosphamide, doxorubicin, vincristine, and prednisone/steroids combination (CHOP), is the standard chemotherapy regime used in patients for aggressive NHL as well as the most used regime for indolent NHL (1, 34). Lymphoma-bearing mice were treated with two cycles of CHOP (CHOPx2) chemotherapy, at days 25 and 35 post-tumor implantation (p.t.i.), as previously described (32). Drug doses used for each chemotherapy cycle were: cyclophosphamide 100 mg/kg i.p., doxorubicin 6 mg/kg i.p., vincristine 0.1 mg/kg i.p., and dexamethasone 0.2 mg/kg i.p. Prophylactic anti-infectious drugs (15 mg/kg of fluconazole and 20 mg/kg of acyclovir) were used during the neutropenia period to avoid potential infections.

Chemotherapy side effects were monitored as before (32), by evaluating body weight changes (BWCs) and hematological toxicity before and after CHOP administration. Lymphocytes, monocytes, and neutrophils recovery post-CHOP were evaluated in an automated blood cell counter and in peripheral blood smears (32).

### *Salmonella* Treatment

**Figure 1:**
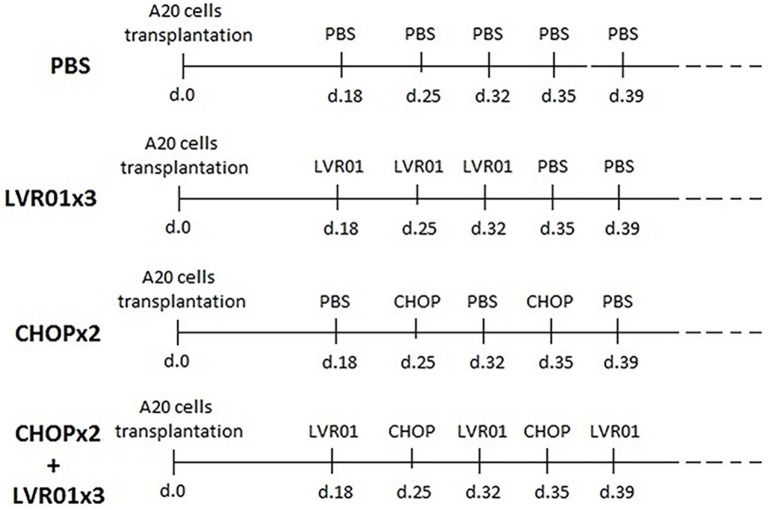
Inoculation scheme. For tumor implantation, 1 × 10^6^ A20 cells were inoculated s.c. at day 0. In two cycles of CHOP (CHOPx2) and CHOPx2 + LVR01x3 groups, chemotherapy cycles were administrated i.p. at days 25 and 35 p.t.i. In LVR01x3 and CHOPx2 + LVR01x3 groups, 1 × 10^6^ CFU of the strain was administrated by i.t. injection on days 18, 25, and 32, and 18, 32, and 39 p.t.i., respectively.

Bacteria were grown in Luria–Bertani medium (Difco Laboratories, Detroit, MI, USA) at 37°C under shaking, to prepare working stock as previously described (25). LVR01 was inoculated by intratumoral (i.t.) injections, with 1 × 10^6^ CFU per tumor in 0.1 ml of PBS. The inoculation scheme for groups PBS, three doses of LVR01 (LVR01x3), CHOPx2, and CHOPx2 + LVR01x3 is shown in Figure 1. This bacteria-administration regimen was decided based on results previously obtained (25). It should be noted that in chemotherapy-treated mice when tumors were not palpable, the bacteria were inoculated subcutaneously in the tumor implantation area.

### Flow Cytometry Analysis of Tumor-Infiltrating Cells

At day 45 p.t.i., five mice from PBS and LVR01x3 groups and five mice with residual lymphoma (partial response) from CHOPx2 and CHOPx2 + LVR01x3 were sacrificed and tumors were removed and prepared to obtain a single-cell suspension. 1 × 10^5^ cells per tumor were immunostained at 4°C in the dark for 30 min with the following antibodies panel: FITC-conjugated anti-CD49b, PECy7-conjugated anti-CD8, APC-conjugated anti-CD3, APCCy7-conjugated anti-CD4, PerCPCy5.5-conjugated anti-CD19, FITC-conjugated anti-CD4, PE-conjugated anti-FoxP3, PECy7-conjugated anti-CD3, APC-conjugated anti-CD25, FITC-conjugated anti-Ly6C, and PE-conjugated anti-Ly6G (all reagents from BD Pharmingen, San Diego, CA, USA). The optimal antibody concentration was defined by titration. For Treg cells analysis, cells were first stained with anti-CD4 and anti-CD25 antibodies, then fixed and permeabilized with a mouse FoxP3 buffer set (BD Pharmingen) and then washed twice with permeabilization buffer and incubated with anti-FoxP3 at 4°C for 30 min in the dark. Flow cytometry data were collected on a FACS Canto II Cytometer (Becton–Dickinson, Oxford, UK). For data acquisition and analysis, FACSDiva (Becton–Dickinson) and Infinicyt (Cytognos, Spain) software were used, respectively.

### Cytokines and Chemokines Determination

Changes in gene expression level of different cytokines and chemokines in the tumor microenvironment were assessed by quantitative reverse transcription-PCR. Mice from PBS and LVR01x3 groups, as well as mice that still had palpable tumors after CHOP treatment (residual lymphoma) from CHOPx2 and CHOPx2 + LVR01x3 groups, were sacrificed at day 45 p.t.i. Tumors were removed and collected in Trizol reagent (Invitrogen, Carlsbad, CA, USA) and stored at −80°C. Tumors were homogenized with an Ultra Turrax homogenizer, and RNA was extracted according to the manufacturer’s instructions (Invitrogen, Carlsbad, CA, USA). Gene expression of the following cytokines and chemokines, *Ccl3, Ccl4, Ccl5, Ccl20, Cxcl1, Cxcl9, Cxcl10, Cxcl11, Cxcl12, Cxcr4, Cxcr7, Il2, Il4, Il6, Il10, Il12, Il17a, Foxp3, Ifng, Lgals1, Tgfb*, and *Tnfa* were assessed.

At day 60 p.t.i., mice (*n* = 5) from CHOPx2 and CHOPx2 + LVR01x3 groups without palpable tumor (complete remission) were sacrificed, and spleens were removed and prepared as a single-cell suspension. For each mouse, equal number of splenocytes (2 × 10^6^ cells/ml) were seeded in complete medium in 24 wells plate and *in vitro* stimulated with either 1 µg/ml of Concanavalin A (ConA) (Sigma-Aldrich), 10 µg/ml of an A20 cell homogenate (A20lys), 10 µg/ml of a B16F1 cell homogenate (B16F1lys) as a non-specific antigen or left non-stimulated as negative control. Cells were incubated at 37°C with 5% CO_2_ for 24 h. Plates were centrifuged and pellets were put in Trizol reagent and stored at −80°C until processed. RNA was extracted according to the manufacturer’s instructions (Invitrogen, Carlsbad, CA, USA) and used to evaluate *Il2, Il4, Il10, Il12, Il17a*, and *Ifng* mRNA levels as follows.

RNA quality and quantity were assessed in a NanoDrop 2000 (Thermo Fisher Scientific, Waltham, MA, USA) by evaluating the ratio at 260/280 nm. Before cDNA synthesis, 1 µg total RNA was treated with DNase I according with the manufacturer’s instruction (Invitrogen). cDNA synthesis was carried out using 1 µl random primers (200 ng), 1 µl dNTPs (10 mM) (Invitrogen), 4 µl buffer (5×), 2 µl 0.1 M DTT (dl-Dithiothreitol), 1 µl RNasa OUT (40 U/µl), 1 µl Moloney murine leukemia virus reverse transcriptase (M-MLV-RT) (200 U) (all reagents from Invitrogen) in a thermocycler using the following steps: 10 min at 25°C, 50 min at 37°C, and 15 min at 70°C. The cDNA was preserved at −20°C. Quantitative PCR was performed using a QuantiTect SYBR green PCR kit according to the manufacturer’s instruction (Qiagen, Hilden, Germany) in a 7900HT RT-PCR System (Applied Bio-systems, Foster City, CA, USA) using the following steps: 15 min at 95°C and 40 cycles of 15 s at 95°C and 1 min at 60°C, with a melting curves analysis at the end. Beta-2-microglobulin (*B2m*) gene was used as housekeeping gene. The primers used are listed in Table 1 and were used at a final concentration of 0.9 µM. The relative mRNA amount in each sample was calculated using the 2^−ΔΔCt^ method (35), where ΔCt = Ct_gene of interest_ − Ct*_B2m_*, and expressed as relative mRNA levels in the test group compared to CHOPX2 control group (fold change).

**Table 1:** Primer sequences used for quantitative RT-PCR.

Gene	Forward primer (5′–3′)	Reverse primer (5′–3′)	Product length (bp)
*B2m*	CCTGCAGAGTTAAGCATGCCAG	TGCTTGATCACATGTCTCGATCC	72
*Ccl2*	CCCTCAACGGAAGAACCAAA	CACATCAGGTACGATCCAGGC	72
*Ccl3*	AACATCATGAAGGTCTCCAC	CCAAGACTCTCAGGCATTCA	294
*Ccl4*	GCCCTCTCTCTCCTCTTGCT	GTCTGCCTCTTTTGGTCAGG	196
*Ccl5*	GGTACCATGAAGATCTCTGCA	AAACCCTCTATCCTAGCTCAT	294
*Ccl20*	TTTTGGGATGGAATTGGACAC	TGCAGGTGAAGCCTTCAACC	69
*Cxcl1*	CTTGGTTCAGAAAATTGTCCAAAA	ACGGTGCCATCAGAGCAGTCT	84
*Cxcl9*	TGGAGCAGTGTGGAGTTCGA	CCTCGGCTGGTGCTGATG	73
*Cxcl10*	GCCGTCATTTTCTGCCTCAT	GCTTCCCTATGGCCCTCATT	127
*Cxcl11*	CAAAATGGCAGAGATCGAGAAA	TGAGCCTTCATAGTAACAATCACTTCA	87
*Cxcl12*	GAAGTGGAGCCATAGTAATGCC	TCCAAGTGGAAAAATACACCG	133
*Cxcr4*	TTCTCATCCTGGCCTTCATC	CTTTTCAGCCAGCAGTTTCC	92
*Cxcr7*	GCCGTACCATTTTGTGGTTC	TGCAACGCTGTAAAGAGCAC	96
*Il2*	CCTGAGCAGGATGGAGAATTACA	CTTTCAATTCTGTGGCCTGCTTGGG	92
*Il4*	ACAGGAGAAGGGACGCCAT	GAAGCCCTACAGACGAGCTCA	95
*Il6*	GTTCTCTGGGAAATCGTGGAAA	AAGTGCATCATCGTTGTTCATACA	78
*Il10*	CATTTGAATTCCCTGGGTGAGA	TGCTCCACTGCCTTGCTCTT	101
*Il12*	ATCACACGGGACCAAACCA	CAGGCAACTCTCGTTCTTGTGTAGT	74
*Il17a*	CTCCAGAAGGCCCTCAGACTAC	GGGTCTTCATTGCGGTGG	69
*Foxp3*	CCCAGGAAAGACAGCAACCTT	TTCTCACAACCAGGCCACTTG	89
*Ifng*	TCAGCAACAGCAAGGCGAAA	CCGCTTCCTGAGGCTGGAT	143
*Lgals1* (galectina-1)	TGAACCTGGGAAAAGACAGC	TCAGCCTGGTCAAAGGTGAT	190
*Tgfb*	GCTGAACCAAGGAGACGGAAT	GAGTTTGTTATCTTTGCTGTCACAAGA	76
*Tnfa*	CATCTTCTCAAAATTCGAGTGACAA	CCTCCACTTGGTGGTTTGCT	63

### Cytokine Protein Production

*In vitro* cytokines protein production was measured in supernatants of splenocytes stimulated as described above. At day 4, plates were centrifuged at 1,200 rpm, and supernatants were stored at −80°C until used. The protein concentration of IL-2, IL-10, IL-12, and IFN-γ were determined by ELISA sets (BioLegend, San Diego, CA, USA), according to the manufacturer’s instructions.

### NK Cytotoxic Assay

At day 60 p.t.i., mice (*n* = 5) from CHOPx2 and CHOPx2 + LVR01x3 groups were sacrificed and spleens were removed, disrupted, and prepared as a single-cell suspension. The unlabeled splenocytes were used as effector (E) cells and YAC-1, sensitive to NK cytotoxicity, served as targets (T) cells. YAC-1 cells were stained with 1 µM of CFSE (Sigma-Aldrich) and placed in a density of 5 × 10^4^/ml in 24-well plate. Splenocytes were put in contact with stained YAC-1 cells in increasing ratios (E:T): 6.25:1, 12.5:1, 25:1, 50:1, 100:1, 200:1 and incubated at 37°C with 5% CO_2_ for 24 h. After incubation, cells were washed and stained with PI for 10 min in the dark at room temperature. Data were acquired by flow cytometry (FACS Canto II, Becton–Dickinson) and Infinicyt (Cytognos, Spain) software was used for the analysis. The cytotoxicity (%) was calculated as: [dead target cells in the sample (%) − spontaneously dead target cells (%)]/[100 − spontaneously dead target cells (%)].

### Antibody Response Assessment

Anti-A20 antibody responses were assessed in sera obtained from peripheral blood as previously described (25). Briefly, ELISA plates were coated overnight at 4°C with 15 μg/well of A20 cell lysate and then washed three times with PBS–0.05% Tween-20 (PBS-T) and blocked with PBS–1% BSA for 1 h. Serum samples diluted 1/50 in PBS–0.05% Tween–1% BSA (PBS-T-BSA) were added in duplicates and incubated for 2 h at 37°C. After the incubation, plates were washed five times with PBS-T. Secondary antibody goat anti-mouse IgG-biotinylated (SouthernBiotech, Birmingham, AL, USA) diluted 1/2,000 in PBS-T-BSA was added and incubated for 2 h at 37°C, followed by streptavidin-PO (Sigma-Aldrich) (to amplify the signal) diluted 1/2500 in PBS-T-BSA. After 45 min of incubation, plates were washed five times with PBS-T. The color was developed using SIGMAFAST OPD tablets (Sigma-Aldrich), following the manufacturer’s instructions.

### Health Condition of Treated Mice

In addition to studying the effect of *Salmonella* i.t. administration, also the impact in the health condition of chemotherapy-treated mice was evaluated (*n* = 10). Clinical and laboratory parameters, including complete blood count, albumin, total protein, bilirubin, aspartate aminotransferase (AST), and alanine aminotransferase were assessed at days 26, 44, and 62 p.t.i. Body condition, grooming, pain, lethargy, diarrhea (gastrointestinal toxicity), and force/stimulus–response were also studied on the same days. The score for each parameter was based on previous reports (36–38). We assigned a general clinical combined score; we choose *0* for an optimal state, *1* presence and *2* exacerbation symptoms/pathology (Table 2). At the same time, hemograms were carried out and body weight change (BWC) was evaluated.

**Table 2:** Overall health status of animals undergoing chemotherapy with or without *Salmonella* treatment.

Clinic parameter	Score	General score
Body condition	0: emaciated	0: optimal state
	1: under conditioned	1: presence of symptoms
	2: well-conditioned	2: exacerbation of symptoms
Grooming	0: correct grooming	0: optimal state
	1: poor grooming	1: presence of symptoms
	2: absence grooming	2: exacerbation of symptoms
Pain	0: absence	0: optimal state
	1: pain when handling	1: presence of symptoms
	2: constantly pain	2: exacerbation of symptoms
Lethargy	0: unrestricted activity	0: optimal state
	1: limited movements	1: presence of symptoms
	2: without movements	2: exacerbation of symptoms
Force/stimulus–response	0: clings to the cage	0: optimal state
	1: clings slightly to the cage	1: presence of symptoms
	2: without clinging	2: exacerbation of symptoms
Diarrhea	0: absence	0: absence
	1: presence	1: presence

*The score for the different clinical parameters is shown. A general score was designated to add these parameters and determine the overall health status*.

### Statistical Analysis

SPSS 17.0 (Statistical Package for the Social Sciences) software for Windows was used to analyze the results. Kaplan–Meier and log-rank test were carried out to determined differences in survival times. Statistical significance of differences between study groups for tumor growth and *in vitro* assays was analyzed using Student’s *t*-test and analysis of variance (ANOVA). A value of *P* < 0.05 was considered statistically significant.

## Results

### *Salmonella* Administration to Animals Undergoing Chemotherapy Delayed Tumor Growth and Prolonged Survival

**Figure 2:**
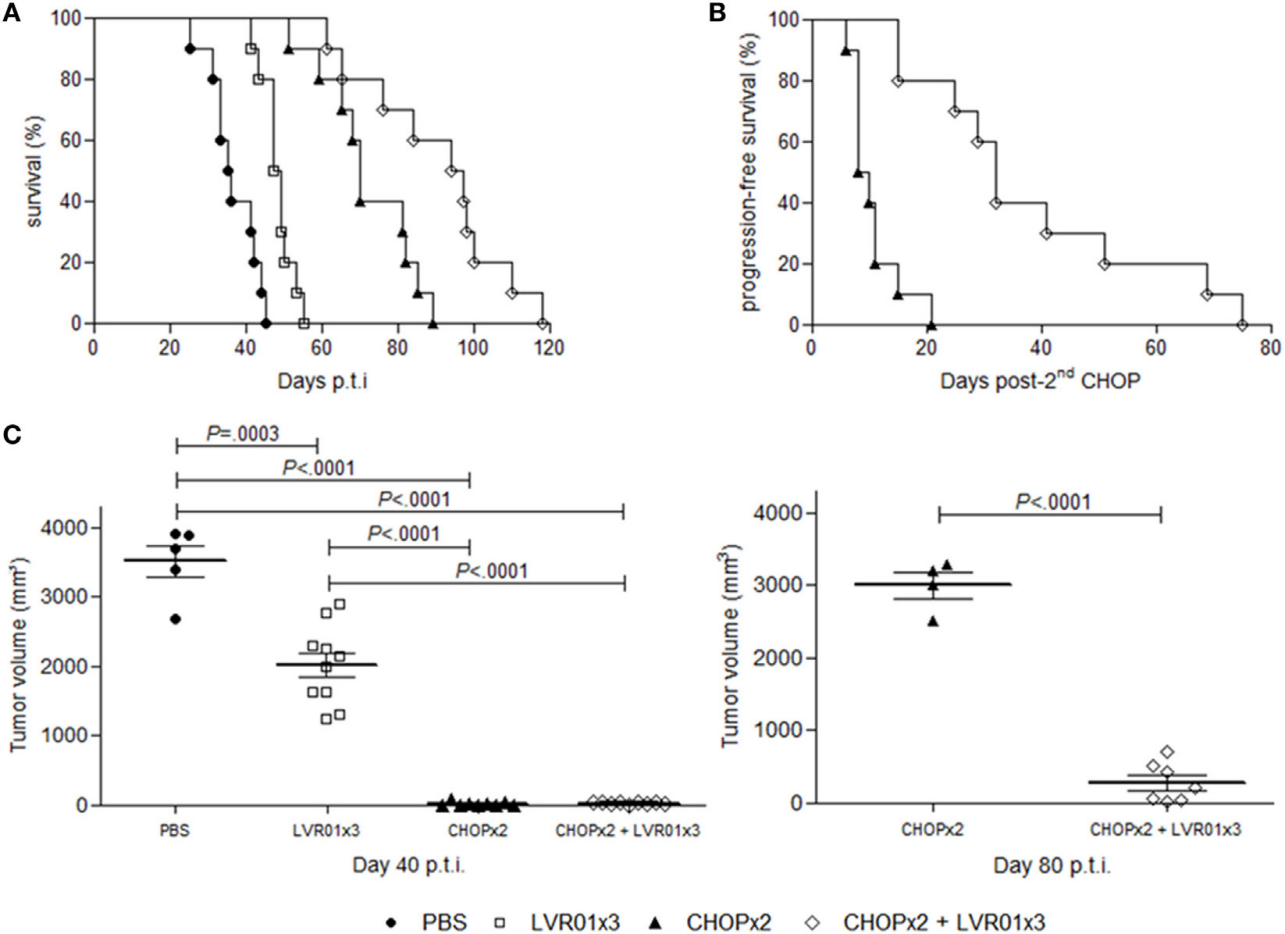
Survival curves and tumor growth. **(A)** Kaplan–Meier plot of mice survival post-tumor challenge. Overall survival was followed up for 120 days (*n* = 10). Significant differences were observed between all groups (log-rank, *P* < 0.0001). **(B)** Progression-free survival post-second CHOP. Significant differences were observed between groups (log-rank, *P* < 0.0001). **(C)** Tumor volume (mm^3^) at day 40 p.t.i. for all groups and 80 p.t.i. for two cycles of CHOP (CHOPx2) and CHOPx2 + LVR01x3 groups. Each dot represents one individual animal. Mean and SD are also depicted. At day 40 p.t.i., significant differences were observed between all groups (*P* < 0.0001, analysis of variance), except groups treated with chemotherapy, for which significant differences are observed at day 80 p.t.i. (*P* < 0.0001, Student’s *t*-test).

The effect of *S*. Typhimurium LVR01 i.t. administration in combination with CHOP chemotherapy on A20-bearing mice was evaluated measuring tumor development and animal survival (Figure 2). Consistently with our previously reported results (25), animals treated with three doses of LVR01 (LVR01x3) in absence of chemotherapy showed a prolonged survival (median OS 48 days) compared with PBS group (median OS 35 days) (*P* < 0.0001, log-rank). Also in line with another previous report (32), among animals receiving only chemotherapy (CHOPx2 group), 92% showed clinic remission of primary tumor for a period of 20 days after second CHOP cycle, after what the primary tumor started to grow again and animals died by primary disease or metastases (median OS 72 days). However, animals receiving both treatments combined (CHOPx2 + LVR01x3 group) had substantially better prognosis than animals receiving either chemotherapy alone (median OS 97 vs. 72 and 48 days for CHOPx2 and LVR01x3 respectively; *P* = 0.009, and *P* < 0.0001, log-rank respectively) (Figure 2A). PFS was also extended (*P* < 0.0001, log-rank) (Figure 2B).

At day 40 p.t.i., animals from groups CHOPx2 and CHOPx2 + LVR01x3 had significantly lesser tumor volume compared to other groups (*P* < 0.0001, ANOVA) (Figure 2C). At that time point tumor volumes of animals receiving CHOP, either alone or in combination with *Salmonella*, were 0 or close to 0 due to the effect of the chemotherapy, irrespectively of *Salmonella* treatment. However, by day 80 p.t.i. animals that received the CHOPx2 + LVR01x3 combination therapy showed significant smaller tumors than those that received CHOP alone (*P* < 0.0001, *t*-test) (Figure 2C).

### Tumor-Infiltrating Leukocytes Were Increased in Mice Receiving Chemotherapy-*Salmonella* Combined Therapy

**Figure 3:**
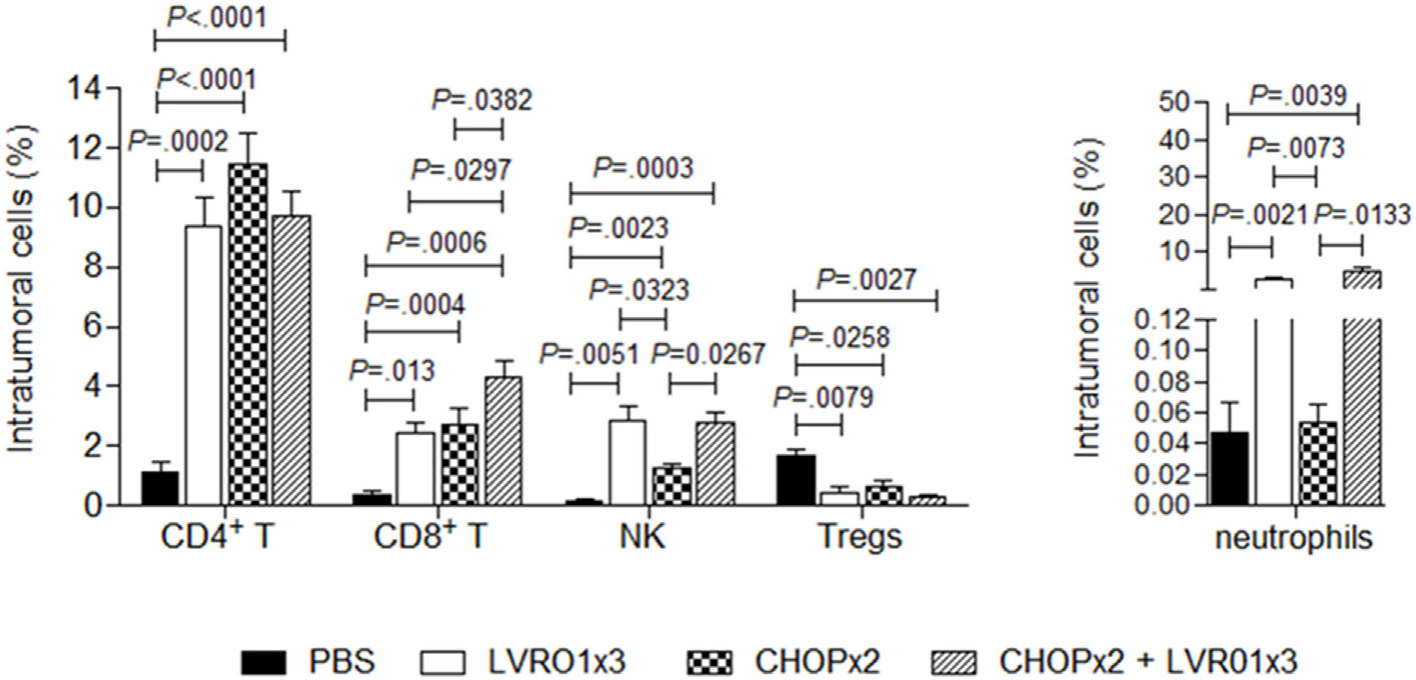
Tumor-infiltrating cells. Mice from PBS, LVR01x3, two cycles of CHOP (CHOPx2), and CHOPx2 + LVR01x3 groups were sacrificed at day 45 p.t.i., and tumors were removed to study tumor-infiltrating cell populations. Percentage of CD4^+^ T lymphocytes (CD3^+^ CD4^+^ cells), CD8^+^ T lymphocytes (CD3^+^ CD8^+^ cells), NK cells (CD3^−^ CD49b^+^ cells), regulatory T cells (Tregs) (CD3^+^ CD4^+^ CD25^+^ FoxP3^+^ cells) and neutrophils (Gr1^+^ CD11b^+^ cells) were determined by flow cytometry. Results are shown as mean ± SD (*n* = 5). Significant differences are shown with *P* value (analysis of variance).

Mice with residual lymphoma after chemotherapy treatment (either alone or in combination with *Salmonella*), as well as mice from PBS and LVR01x3 groups, were sacrificed at day 45 p.t.i. and tumors were removed and processed for analysis of tumor-infiltrating cell populations. Intratumoral CD4^+^ T cells percentages were significantly higher in all treated groups (i.e., LVR01x3, CHOPx2, and CHOPx2 + LVR01x3) compared to non-treated control group (PBS) (*P* < 0.0001, ANOVA) (Figure 3). Conversely, percentages of regulatory T cells (Treg) were decreased in all treated groups as compared to control group (*P* = 0.05). In all cases, there were no differences in these cell populations between groups receiving different treatments. Instead, percentages of intratumoral CD8^+^ T cells were significantly increased in animals receiving the combined therapy (CHOPx2 + LVR01x3) as compared to mice from all other groups (*P* < 0.0001) (Figure 3).

On the other hand, animals receiving *Salmonella* treatment (either alone or in combination with chemotherapy) showed a similar increase in intratumoral neutrophils and NK cells as compared to control group. Chemotherapy also induced an increase in intratumoral NK cells although significantly lower than *Salmonella* therapy (Figure 3).

### The Combined Therapy Induced the Expression of a Broad Array of Cytokines and Chemokines in the Tumor Microenvironment

**Figure 4:**
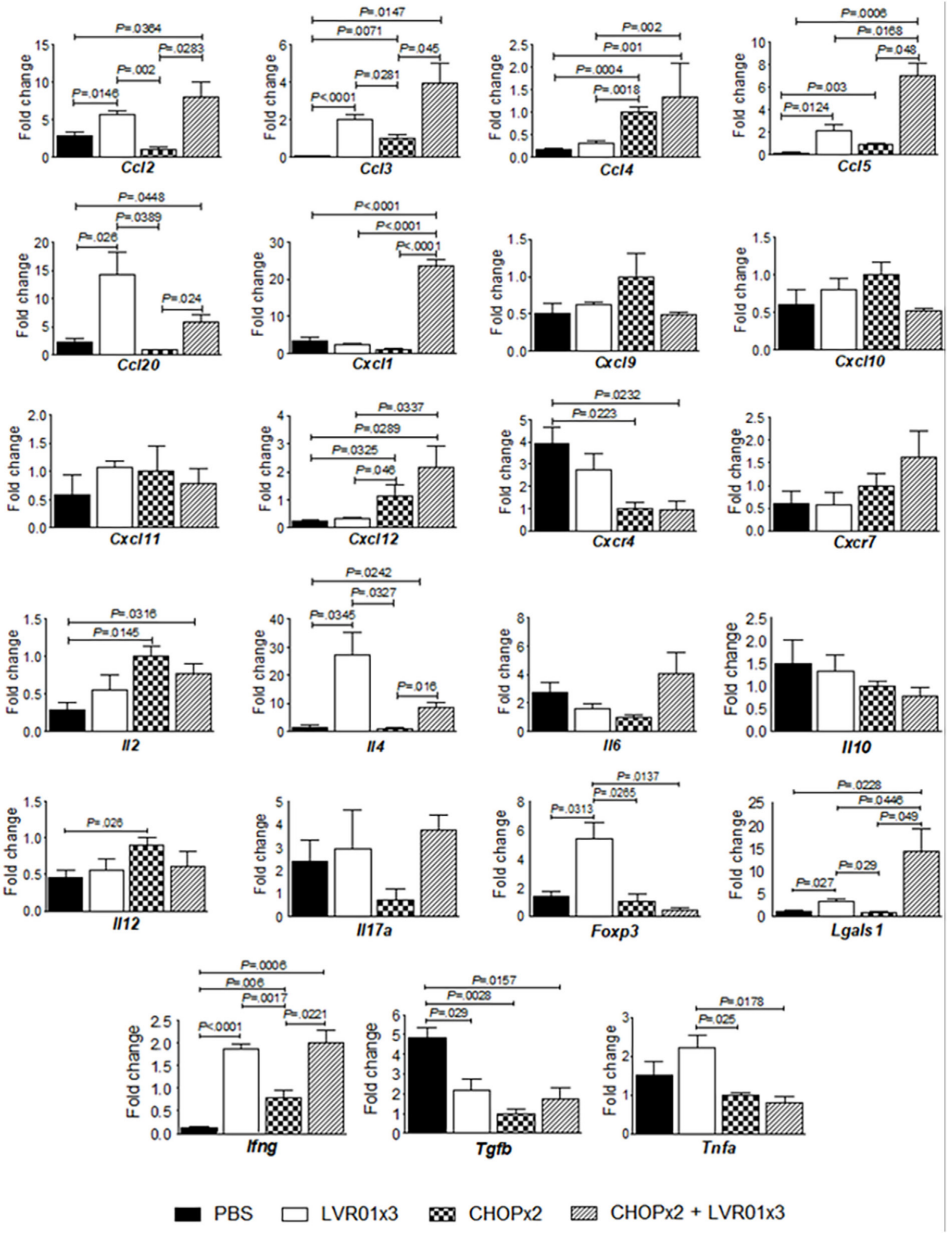
Cytokines/chemokines gene expression levels in the tumor microenvironment. Mice from PBS, LVR01x3, two cycles of CHOP (CHOPx2), and CHOPx2 + LVR01x3 groups were sacrificed at day 45 p.t.i., and tumors were removed to assess the expression of cytokine and chemokines genes by quantitative RT-PCR on total tumor RNA. Gene mRNA values were normalized to that of *B2m* mRNA, and the results were expressed relative to mRNA levels in the CHOPx2 group for each day (fold change). Results are shown as the mean ± SD (*n* = 5). Significant differences are shown with *P* value (analysis of variance).

Analysis of cytokines and chemokines mRNA levels in tumor from mice with residual lymphoma was carried out at day 45 p.t.i. As shown in Figure 4, the combined treatment induced a cytokine/chemokine gene expression profile in the tumor microenvironment that closely resembles each treatment alone, but with some differences. Animals that received either chemotherapy, *Salmonella*, or both, showed upregulation of *Ccl3* (*P* = 0.0008, ANOVA), *Ccl5* (*P* < 0.0001), and *Ifng* (*P* < 0.0001) and downregulation of *Tgfb* (*P* = 0.0031) as compared to untreated mice. Chemotherapy treatment also augmented *Ccl4* (*P* < 0.0001), *Cxcl12* (*P* = 0.0115), and *Il2* (*P* = 0.05) and downregulated *Cxcr4* (*P* = 0.0225) gene expression, whereas *Salmonella* treatment upregulated the expression of *Ccl2* (*P* = 0.0039), *Ccl20* (*P* = 0.0108), *Lgals1* (*P* = 0.004), and *Il4* (*P* = 0.0072). However, only the combined treatment induced also upregulation of *Cxcl1* gene expression (*P* < 0.0001). On the other hand, chemotherapy induced upregulation of *Il12* but *Salmonella* administration counteracted this effect in the combined therapy. Conversely, *Salmonella* induced upregulation of *Tnfa* and *Foxp3* gene expression, which was not maintained with the combined treatment (Figure 4).

### Systemic Antigen-Specific Responses Were Induced in Mice Treated with the Combined Therapy

**Figure 5:**
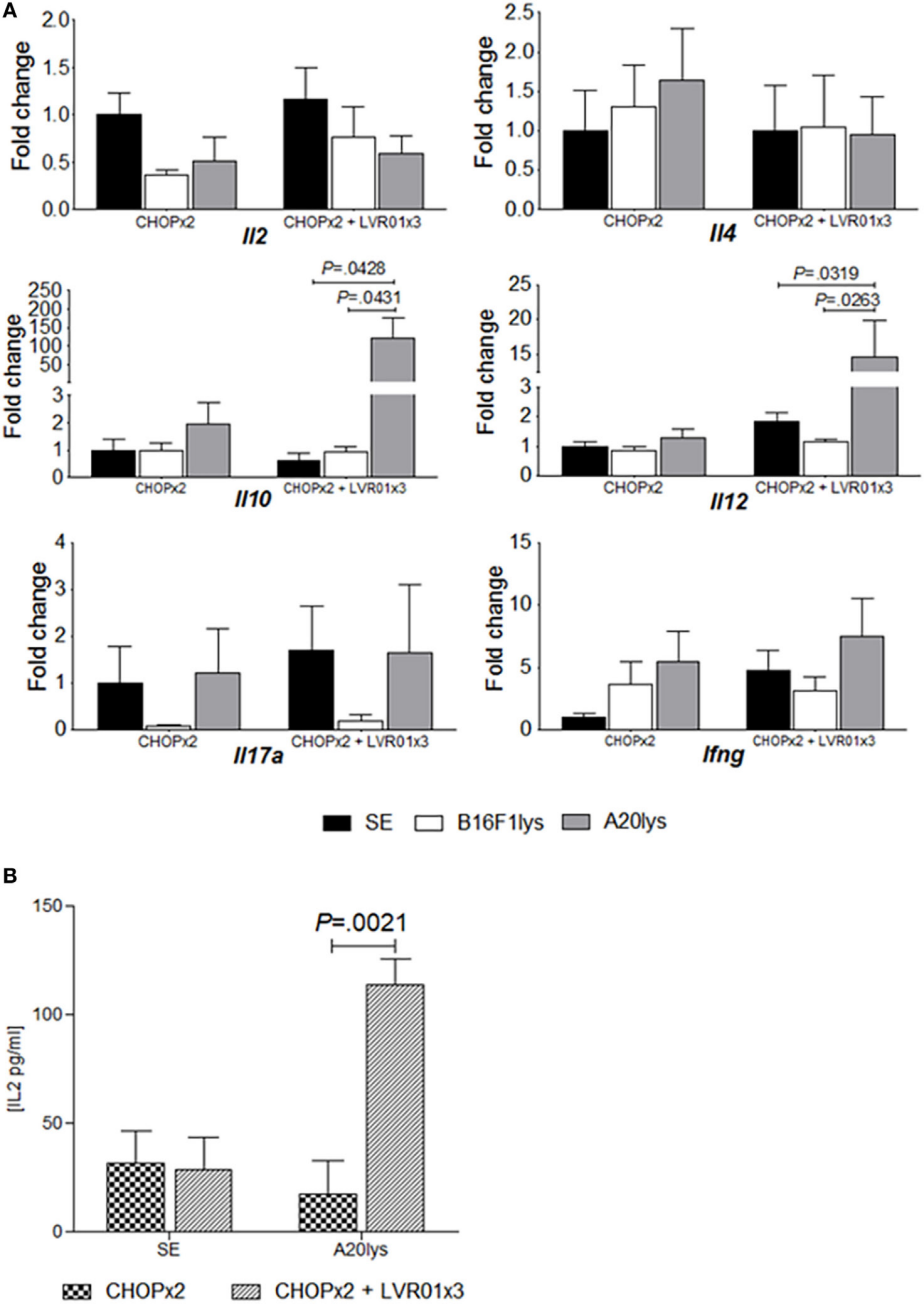
Antigen-specific cytokine responses in stimulated splenocytes. **(A)** Cytokines gene expression levels in stimulated splenocytes. Mice from two cycles of CHOP (CHOPx2) and CHOPx2 + LVR01x3 groups were sacrificed at day 60 p.t.i., and spleens were removed to study the expression of cytokine genes by quantitative RT-PCR in stimulated splenocytes. Gene mRNA values were normalized to that of *B2m* mRNA, and the results were expressed relative to mRNA levels in non-stimulated condition from CHOPx2 group (fold change). Results are shown as the mean ± SD (*n* = 5). A significant increase in *Il10* and *Il12* gene expression for antigen-specific stimulated splenocytes (A20lys) was observed. Significant differences are shown with *P* value [analysis of variance (ANOVA)]. **(B)** Cytokines concentration in the supernatant of stimulated splenocytes at day 70 p.t.i. IL-2 concentration values (picograms per milliliter) are shown as the mean ± SD (*n* = 5) for non-stimulated and stimulated with A20lys. Significant differences are observed between groups when splenocytes were stimulated with tumor-specific antigen (A20lys) (*P* = 0.0021, ANOVA).

At day 60 p.t.i. mice from CHOPx2 and CHOPx2 + LVR01x3 groups were sacrificed, and spleens were removed and processed to assess cytokines mRNA expression levels upon antigenic stimulation. As shown in Figure 5, a significant increase in *Il10* and *Il12* gene expression was observed in animals receiving the combined therapy upon stimulation with an A20 cell lysate (A20lys), but not in mice receiving chemotherapy alone (*P* = 0.0066, *P* = 0.0016, ANOVA for each gene, respectively) (Figure 5A). This response was antigen-specific since stimulation with an unrelated antigen (B16F1 lysate) did not produce any modification in gene expression.

Cytokine concentrations in the supernatant of stimulated splenocytes from CHOPx2 and CHOPx2 + LVR01x3 groups were also measured by ELISA. As shown in Figure 5B, IL-2 production was observed in splenocytes from CHOPx2 + LVR01x3 mice upon specific antigen A20lys (*P* = 0.0021, Student’s *t*-test), but not in splenocytes from mice of the CHOPx2 group. IL-10, IL-12, and IFNγ were also measured in the same supernatants, but their concentrations were below the detection.

### Combined Immunotherapy Induced an Increase in NK Cell-Mediated Cytotoxicity

**Figure 6:**
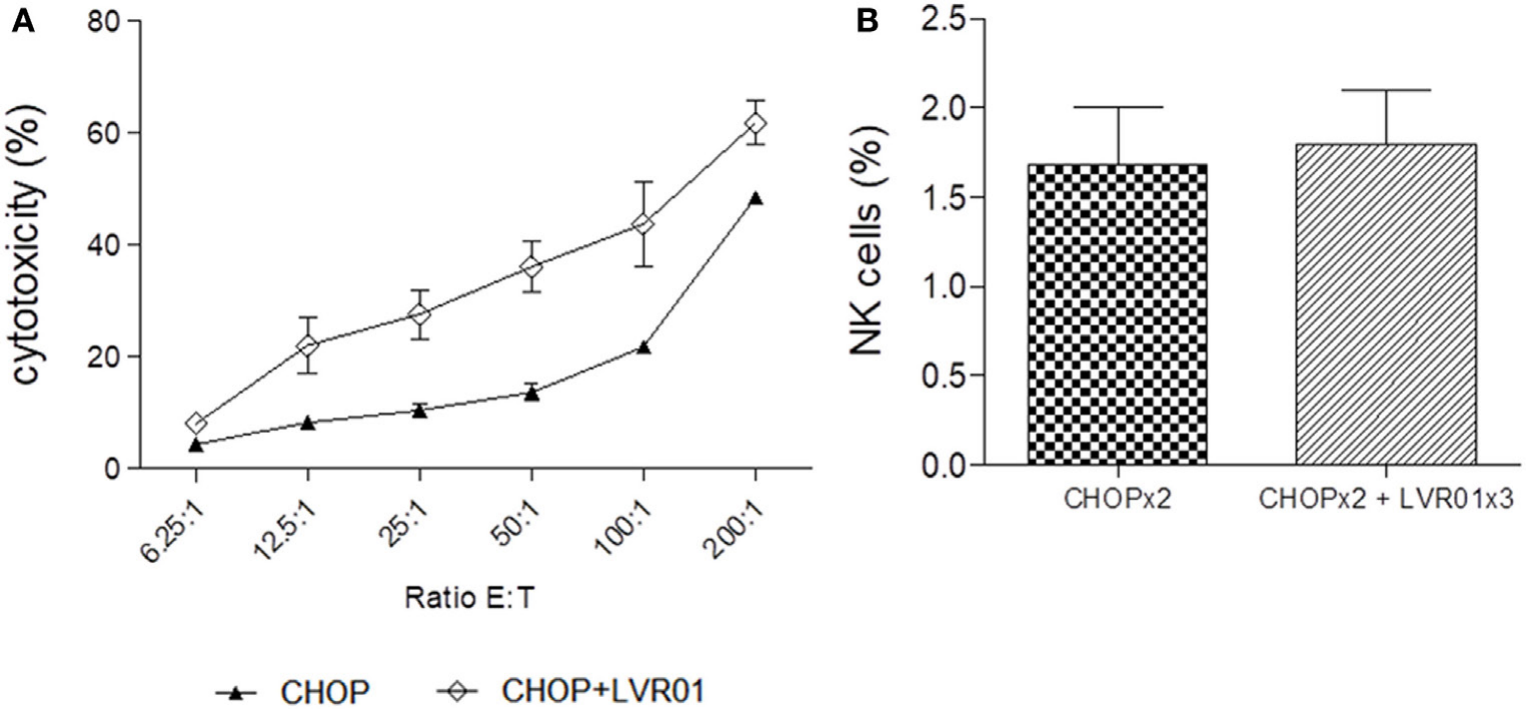
Cytotoxicity. **(A)** NK cell-mediated cytotoxicity percentage at different ratios E:T for two cycles of CHOP (CHOPx2) and CHOPx2 + LVR01x3 is shown. Splenocytes from mice sacrificed at day 63 p.t.i. were used as effector cells and YAC-1 as target cells. Significant differences in all ratios were observed between both groups (*P* < 0.0001, Student’s *t*-test). Results are shown as the mean ± SD (*n* = 5). **(B)** Spleen NK cells percentage of CHOPx2 and CHOPx2 + LVR01x3 groups are shown. No significant differences were observed between groups.

Splenocytes taken at day 60 p.t.i. were also used as effector cells for the NK cell-mediated cytotoxicity assay, using the NK cytotoxicity-sensitive YAC-1 cell line. A significant increase in cytotoxic activity of splenocytes derived from CHOPx2 + LVR01x3 treated mice was observed in all E:T ratios (*P* < 0.0001, Student’s *t*-test) (Figure 6A). Importantly, the percentage of NK cells within splenocytes remained the same for both groups (Figure 6B). Therefore, the increase in cytotoxicity could be attributed to an increase in the cytotoxic capacity of NK cells.

### *Salmonella* Treatment Induced Anti-A20 Specific Humoral Immune Responses in CHOP-Treated Animals

**Figure 7:**
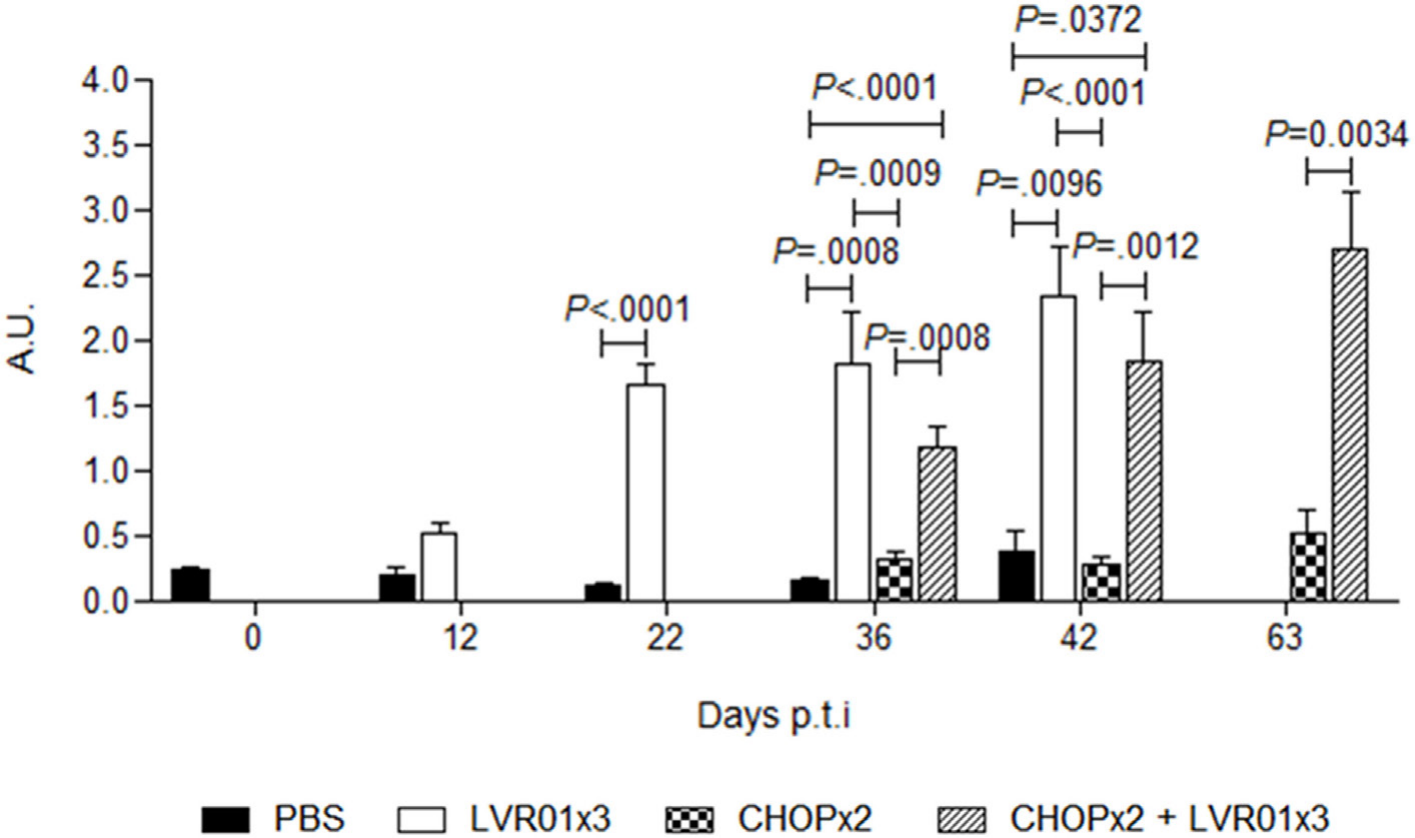
Humoral responses against A20 lymphoma cells. Anti-A20 lymphoma IgG levels were determined in serum by ELISA at days 12, 22, 36, and 42 p.t.i. for PBS and LVR01x3 groups, and at days 36, 42, and 63 for two cycles of CHOP (CHOPx2) and CHOPx2 + LVR01x3 groups. IgG basal levels were determined in naïve mice at day 0 p.t.i. Data are shown as mean of arbitrary units (A.U.) ± SD (*n* = 5). At days 36 and 42 p.t.i. LVR01x3 and CHOPx2 + LVR01x3 developed a significantly higher anti-A20 IgG antibody response compared with PBS and CHOPx2 groups; at day 63 p.t.i. significant differences between CHOPx2 and CHOPx2 + LVR01x3 groups still remained. Significant differences are shown with *P* value (analysis of variance, Student’s *t*-test).

Sera obtained from peripheral blood at days 12, 22, 36, and 42 p.t.i. for all groups and 63 p.t.i. from CHOPx2 and CHOPx2 + LVR01x3 mice (*n* = 5) were assayed by ELISA to determine the anti-A20 lymphoma humoral response. Animals receiving LVR01, either with or without chemotherapy, showed significantly higher levels of IgG anti-A20 compared with untreated groups from day 22 p.t.i. thereafter (Figure 7).

### *Salmonella* Treatment Decreases Chemotherapy Toxicity

**Figure 8:**
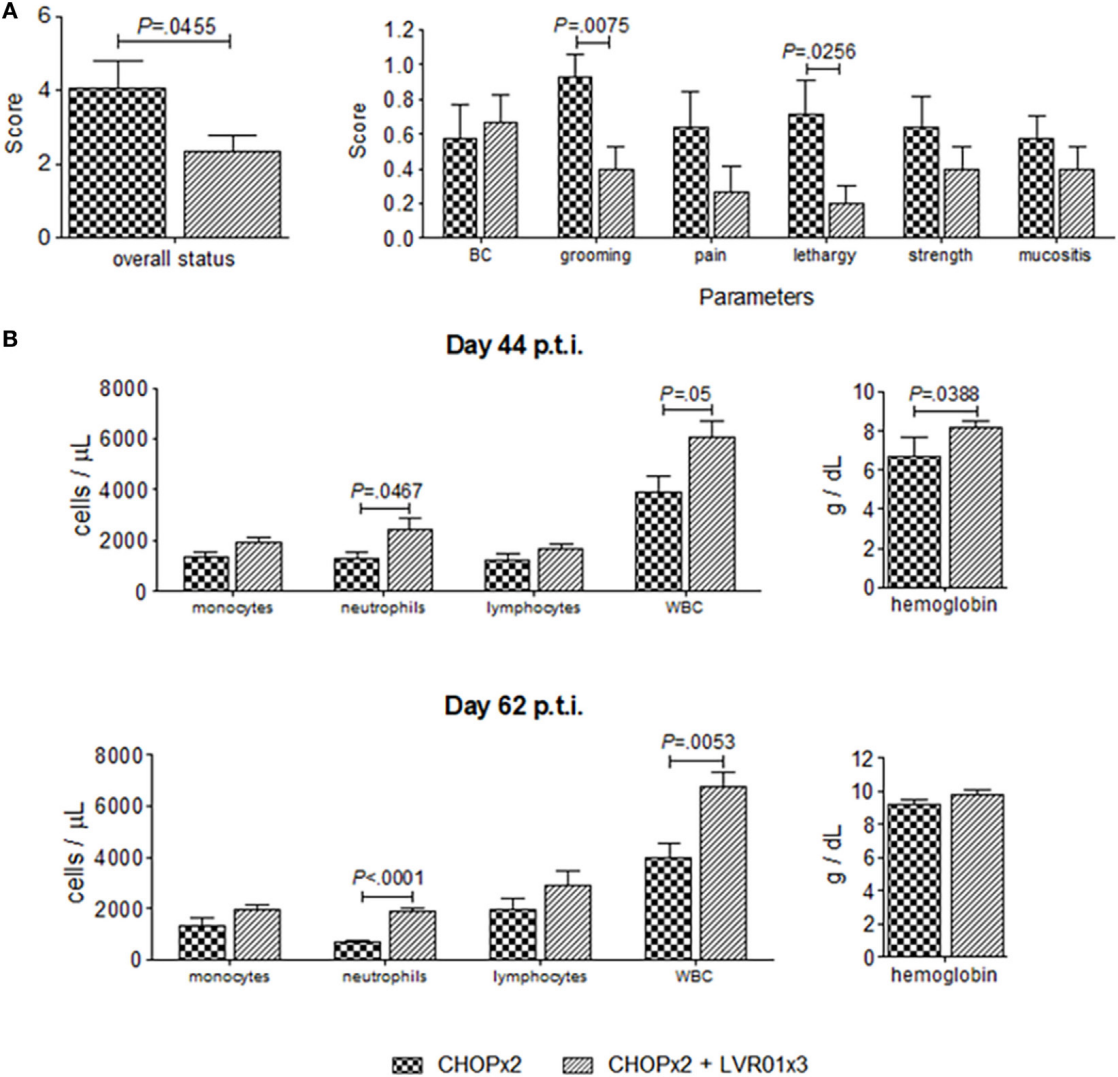
Overall health status of animals undergoing chemotherapy with or without *Salmonella* treatment. **(A)** Overall status and clinical parameters used to determine it at day 44 p.t.i. for two cycles of CHOP (CHOPx2) and CHOPx2 + LVR01x3 groups. Data are shown as the mean ± SD (*n* = 10). **(B)** Blood cells count and hemoglobin concentration ± SD (*n* = 5), obtained from hemograms performed at days 44 and 62 p.t.i. Significant differences are shown with *P* value (Student’s *t*-test).

The effect of *Salmonella* administration in the overall health condition and behavior of chemotherapy-treated mice was evaluated using clinic and laboratory parameters. At day 44 p.t.i., significant differences were observed in grooming (*P* = 0.0075, Student’s *t*-test) and lethargy (*P* = 0.0256), resulting in an improved overall health status of animals undergoing chemotherapy treated with *Salmonella* (*P* = 0.0455) (Figure 8). These differences were accompanied by differences in laboratory parameters, where a significant increase in the number of white blood cells (WBC) and neutrophils, and an increase in the hemoglobin concentration was observed in animals with the combined therapy (CHOPx2 + LVR01x3) compared with CHOPx2 group (*P* = 0.05, *P* = 0.0467, and *P* = 0.0388, respectively) (Figure 8). Differences in the number of WBC and neutrophils were still observed at day 62 p.t.i.

## Discussion

In this work, we demonstrated that *Salmonella* immunotherapy could be administered to lymphoma-bearing animals that are undergoing chemotherapy treatment without any deleterious effects. Instead, *Salmonella* administration resulted in improvement of overall health status of animals receiving chemotherapy, suggesting that it reduces chemotherapy toxicity. Previous reports from others have shown that *Salmonella* treatment improves cyclophosphamide antitumor effect in a murine melanoma model and that when combined with cisplatin it enhances antitumor immune responses in a murine lung tumor and hepatoma (39, 40). *Salmonella* has also been assessed in combination with the chemotherapy drug 5-fluorouracil in a colorectal metastatic model (41). Here, we applied CHOP, the standard four drugs combination used in the clinics, to A20-bearing mice and demonstrated that administration of three doses of *Salmonella* LVR01 in combination with CHOP induced significantly higher activity than each treatment alone. The main features of the immunity elicited were infiltration of the tumor site with immune cells together with overexpression of key cytokines and chemokines in the tumor microenvironment and the development of tumor-specific systemic immune responses.

The improved effect induced by the combined treatment was the result of maintaining the effects of each treatment alone sometimes with an additive effect, plus a few features that only appeared when the treatments were applied together. Any of the treatments (LVR01x3, CHOPx2, and CHOPx2 + LVR01x3) induced a significant increase of tumor-infiltrating CD4^+^ T lymphocytes and a decrease in Treg cells population as compared to untreated control (PBS). Further, a significant increase of *Il2* and *Ifng* gene expression was also observed in all treated groups; IL-2 and IFN-γ are key cytokines to develop effective immune responses (42–44), and the tumor-infiltrating CD4^+^ T cells are most likely one of the source of these cytokines. The decrease in the number of Tregs cell in LVR01-treated mice is in accordance with the findings that *Salmonella* induces a decrease in immunosuppressive cell populations that promote the change from an immunosuppressive to an immunogenic tumor microenvironment (17, 45). In CHOPx2 group, the numbers of Tregs were also lower than PBS group, probably due to the administration of cyclophosphamide as part of CHOP regime, since some chemotherapeutic drugs, including cyclophosphamide, has been reported to suppress Treg cell population, enhancing immunity (46, 47).

A marked increase in tumor-associated neutrophils was observed in animals receiving *Salmonella*, which is in line with our previous report showing that LVR01 i.t. administration increases the number of neutrophils into tumor site with therapeutic benefits (25). Vendrell and colleagues also described that neutrophils are the main tumor-infiltrating cells after intratumoral treatment with *Salmonella* Typhi in a T-cell lymphoma preclinical model (48). In addition, we observed a significant increase in *Ccl3* gene expression in *Salmonella*-treated groups, a chemokine produced by macrophages whose main function is to recruit and activates neutrophils (49, 50).

Tumor-infiltrating NK cells were increased in animals that received LVR01 either alone or together with CHOP. Accordingly, we also found in these groups a significant increment in *Ccl2, Ccl3*, and *Ccl5* gene expression which are chemokines involved in the recruitment of NK cells (51). Further, NK cells from these groups of animals had a significant increment in cytotoxic activity. NK is a heterogeneous cell population (cytotoxic, immunoregulatory, and memory cells) which have been described as critical effectors in tumor immunology with attributes of both innate and adaptive immunity (52, 53). Moreover, recent clinical data provide evidence of NK cell activity against hematologic malignancies, which could be translated into feature therapeutic strategies (54, 55).

In addition to *Salmonella*- and CHOP- only induced effects, we also found that the combined therapy significantly increased the number of CD8^+^ T cells recruited to tumor site together with a significant increment in *Ifng* gene expression as compared with all the other groups. Effector CD8^+^ T cells commonly represent the majority of tumor-infiltrated lymphocytes in tumors with good prognosis (56).

We have previously demonstrated that live *Salmonella* LVR01 induces cell death on A20 cells, as well as upregulation of co-stimulatory molecules in A20 and human lymphoma cells (24). Further, we showed that LVR01 administered to A20-bearing animals persisted at the tumor site for up to 25 days and induced the recruitment of T and NK cells with a clear activated phenotype (25).

Altogether, these results suggest that *Salmonella* immunotherapy potentiates the response following chemotherapy by several combined mechanisms of action, which includes a direct tumoricidal activity, together with a general immune activation status, inducing the expression of co-stimulatory molecules in tumor cells that thus become more efficient antigen-presenting cells (APC), recruitment of activated immune cells to the tumor site, expression of pro-inflammatory mediators, and the development of a systemic antitumor immune response that includes development of specific antibody response and NK cytotoxicity.

Increasing evidence support the idea that chemokines and their receptors are key players in the interaction between tumor and stroma, either creating a permissive microenvironment for tumor growth and metastasis, or an immunogenic microenvironment that prevents or delay tumor development (50, 57–62). For example, in melanoma, the upregulation of chemokine genes, such as *Ccl2, Ccl3, Ccl4, Ccl5, Cxcl9*, and *Cxcl10*, in the tumor microenvironment correlates with the influx of activated T cells into the tumor supporting the antitumor immune response (60). On the contrary, it has also been reported low amount of these chemokines in poorly infiltrated melanomas and colorectal carcinomas (60–62). Our results showed that *Salmonella* LVR01 plus CHOP chemotherapy induces a significant increase in gene expression of several chemokines at day 45 p.t.i., which it is consistent with the increased number of tumor-infiltrating cells (neutrophils, NK cells, and CD4^+^ and CD8^+^ T cells,) in those mice. The increase in the number of tumor-infiltrating NK cell in *Salmonella*-treated group is consistent with the increment in *Ccl2, Ccl3*, and *Ccl5* gene expression observed in these groups, since one function of these chemokines is the recruitment of NK cells (51). By contrast, the implication of this chemokines in different pathologies related with the immune system, including cancer, it has been described (63). Further, the significant increment of *Ccl3* gene expression is consistent with the increased number of neutrophils since this chemokine, produced mostly by macrophages, is implicated in the recruitment and activation of this cell population (49, 50). CCL3 has emerged as a potent activator of both innate and adaptive responses, playing a critical role in recruiting of immune cell population into the tumor. This chemokine is an essential player in regulating lymph node homing of DC subsets, and orchestrating T cell APC. Besides, CCL3 participates in the proliferation of hematopoietic stem/progenitor cells (64). Another neutrophil-attractant chemokine is CXCL1, expressed by neutrophils, macrophages, and epithelial cells. The increase of this chemokine was observed only in the group treated with the combined therapy. CXCL1 has been described to participate in angiogenesis, inflammation, and tumorigenesis processes (65–67). In addition, we found a significant increase in *Cxcl12* expression in CHOP-treated group but not in its receptor *Cxcr4*, being relevant, since the CXCL12–CXCR4 axis has been associated with tumor invasion and metastases (57). Instead, an increase in *Cxcr4* gene expression in PBS group was observed, in accordance with the fact that this chemokine receptor has been shown overexpressed in several human cancers (42).

An interesting founding was the significant increment in galectin-1 (*Lgals1*) gene expression in the CHOPx2 + LVR01 group. Galectins are a family of protein that binds to β-galactoside sugars. These carbohydrates are implicated in cell–cell adhesion, cell–matrix interaction, immune system homeostasis, and cell growth (68). It has been described that galectin-1 is upregulated in different tumors, promoting the tumor immune scape, promoting T cell apoptosis, angiogenesis, and transformation (69–72). Particularly, the role of galectins has been described in hematological neoplasias, observing an association between increased gene expression of galectins and tumor progression (73). Perhaps, the significant increment in *Lgals1* gene expression is due to the fact that the animals are in the suboptimal condition (residual lymphoma). However, no decrease in T lymphocytes was found as a possible effect of galectin-1, but an increase was observed in this population. Further studies would be needed to determine the role of this protein in the antitumor activity due to the *Salmonella* therapy.

Another important group of players in tumorigenesis with influence in cancer prognosis are cytokines (44). *Salmonella* induced upregulation of *Il2, Il4, Ifng*, and *Tnfa* gene expression in the tumor, which might be involved in the intratumoral recruitment of CD4^+^ T, CD8^+^ T, and NK cells. Further, we observed in all treated groups downregulation of *Tgfb* gene expression, which has been described as one of the principal immunosuppressive factor secreted by tumor cells, creating a local environment of immune tolerance (42).

*Salmonella* immunotherapy combined with CHOP in A20-bearing mice elicited tumor-specific immune responses, demonstrated as A20 antigen-specific splenocytes proliferation and anti-A20 IgG antibodies. Previously, we reported that LVR01 treatment induced an antigen-specific humoral response in A20-bearing mice (25), and here, we demonstrated that this response is not abrogated in animals that have received CHOP treatment. In lymphomas, antibody-mediated effector responses are very important, with anti-CD20 monoclonal antibodies as part of the standard treatment for NHL (4, 74). These antibodies can elicit direct antitumor effect *via* apoptosis or other cell death pathways, antibody-dependent cellular cytotoxicity, and complement-dependent cytotoxicity, mediated by Fc receptors present in NK cells and macrophages (74).

Preclinical models based on subcutaneously injected tumor cells, although extensively used in preclinical studies, still do not fully resemble the human condition. Human NHL are highly heterogeneous diseases, half of which are diffuse large B-cell lymphomas (DLBCL), followed in prevalence by follicular lymphomas, marginal zone lymphomas, and others. The A20 lymphoma model despite being a transplantable tumor implanted subcutaneously still has several features that make it a widely used model (75). Among them, tumors are histologically and biologically similar to human DLBCL (most frequent NHL), with large tumor masses and lymphoid nodes involvement and without bone marrow infiltration (76), and it uses immune competent mice, thus is appropriate for immunotherapies studies.

Taken altogether, our results demonstrate that *Salmonella* immunotherapy applied to CHOP-treated NHL mice elicit strong and specific markers of immunity that leads to a notable reduction of tumor activity with a marked survival benefit over either therapy alone. *Salmonella* stimulates both innate and adaptive immune response, with immune cell population’s influx to the tumor site, an increased chemokines and pro-inflammatory cytokines gene expression, activation of NK cells, a systemic antigen-specific response, and an anti-A20 specific humoral response compared with control group. Chemotherapy treatment exerts antitumoral activity by direct cytotoxic effect and promoting an immunogenic microenvironment, enhancing the response produced by LVR01 administration. Since CHOP is one of the most used chemotherapy regimens for NHL and given the excellent safety profile of LVR01, this strategy could be an effective therapy against B-cell lymphoma, to be transferred into clinical trials.

## Ethics Statement

All protocols for animal experimentation were carried out in accordance with procedures authorized by the University’s Ethical Committee for Animal Experimentation, Uruguay, to whom this project was previously submitted.

## Author Contributions

SG and JC conceived and supervised the project, designed the experiments, and analyzed the data. TB designed and performed the experiments and analyzed data. MM participated in the *in vivo* experiments and analysis of the data. TB, SG, and JC wrote the manuscript. All authors contributed to enriching discussions. All authors read and approved the final manuscript.

## Conflict of Interest Statement

The authors declare that the research was conducted in the absence of any commercial or financial relationships that could be construed as a potential conflict of interest.
